# Autocollimation-Based Roll Angle Sensor Using a Modified Right-Angle Prism for Large Range Measurements

**DOI:** 10.3390/s25041250

**Published:** 2025-02-18

**Authors:** Yan Guo, Yu Zhang, Jiali Ji, Huige Di, Qing Yan, Li Wang, Dengxin Hua

**Affiliations:** School of Mechanical and Precision Instrument Engineering, Xi’an University of Technology, Xi’an 710048, China; guoyan@xaut.edu.cn (Y.G.); 2230221154@stu.xaut.edu.cn (Y.Z.); 2220220088@stu.xaut.edu.cn (J.J.); dihuige@xaut.edu.cn (H.D.); yanqing@xaut.edu.cn (Q.Y.); wlfuyun@xaut.edu.cn (L.W.)

**Keywords:** roll angle, autocollimation, large range measurements, right-angle prism

## Abstract

An autocollimator is a popular angle measuring apparatus which lacks the capability to measure the roll angle. This paper proposes a novel roll angle sensor with a large measuring range that is based on the autocollimation principle. A modified right-angle prism (MRP) functions as a reflector to admit a collimated beam and return two outgoing beams to the sensor head. The roll angle of the MRP can be attained by analyzing the moving tracks of the two light spots focused on a photodetector. The mathematical model is derived in detail, and the experimental results show that the measuring accuracy of the proposed sensor is ±13.85 arcsec over a range of 360°. These results verify the feasibility of the proposed sensor for roll angle measurements that require a large measuring range.

## 1. Introduction

Roll angle measurements, which determine the rotation of an object around the direction of its motion, play an important role in monitoring the condition of large facilities [1,2], the attitude control of flying targets [3,4], and the error correction of machine tools [5,6]. In recent years, various roll angle measurement methods based on different structures and principles have emerged [7,8,9]. Among these, optical methods are more accurate and have gradually become a popular research topic. However, the actual measurement of the roll angle remains a challenge owing to the peculiarity of in-plane rotation.

Several research teams have conducted extensive studies on this issue. Zhang et al. [10] proposed a polarization method in which a linearly polarized beam passed through a half-wave plate (HWP) and several polarizing optical elements and then became an elliptically polarized beam. The roll angle of the HWP was measured by detecting the beam ellipticity. Zhai et al. [11] presented a scheme that adopted two quadrant detectors (QDs) to measure the vertical displacements of a reflected beam and a transmitted beam generated by a standard right-angle prism, and they determined the roll angle of the prism based on a computation model. Cai et al. [12] designed a roll angle measurement system whose sensor head emitted two parallel laser beams and whose moving unit containing two QDs translated along a guideway and received both beams. The measured straightness error could be transformed into the roll angle of the moving unit. Lee et al. [13] proposed a technique that utilized the birefringence effect of crystals to decompose s- and p-polarized beams and employed a polarization camera to monitor the phase difference between both beams caused by the rolling crystal to obtain its roll angle. Kumar et al. [14] produced a lateral shearing device to shear an incident beam into two orthogonally polarized beams and make them interfere. The roll angle of this device could be determined by measuring shifts in the interference fringes. Hsieh et al. [15] developed a six-degree-of-freedom interferometer in which three detecting units sensed the displacements of a positioning stage along the *x*-, *y*-, and *z*-axis. Through pairwise comparison, the three-dimensional angles of the stage, including the roll angle, were attained. Liu et al. [16] presented a holographic goniometry that employed two laser beams to symmetrically irradiate a measured object. Then, two reflected beams and one reference beam formed a hologram, from which the roll angle of the object could be extracted. Kim et al. [17,18] constructed a grating-based measurement system where an incident beam illuminating the grating yielded 0th and ±1st order diffraction beams. The grating’s roll angle was calculated from the coordinates of these beams received by photodetectors. Gao, Ren, Li, and Guo [19,20,21,22,23] ameliorated the classical autocollimation method by replacing the plane mirror with a customized grating, prism, or mirror assembly, the roll angle of which could be calculated based on the position and obliquity variations of the autocollimation images. While these existing methods have made improvements to the process of roll angle measurement, some limitations remain. Firstly, these methods can achieve a resolution of up to 0.05 μrad [15] or 0.01 arcsec [21] and a measuring accuracy of ±0.2 arcsec [20], but the measuring range merely reaches ±20° [22], which hampers their widespread applications. Secondly, some methods require sophisticated system architectures and data processing algorithms, which are costly and slow in measuring speed.

Considering the simple structure and convenient implementation of the autocollimation method, this paper proposes a roll angle sensor with a large measuring range based on the autocollimation principle. A modified right-angle prism (MRP) replaces the plane mirror to admit a collimated beam and return two outgoing beams, which then converge into two light spots on a photodetector. The roll angle of the MRP can be determined by analyzing the moving tracks of both light spots. Compared to the existing techniques, the proposed roll angle sensor features a measuring range of up to 360°, and it has a faster measuring speed than the methods using image-acquisition photodetectors to collect holograms, interference fringes, and multiple autocollimation images owing to the collection of only two light spots. The framework of this paper are as follows: The optical system and measurement method are detailed in Section 2. The experimental process and result analysis are described in Section 3. Finally, the conclusions and related discussions are presented in Section 4.

## 2. Methodology

### 2.1. Optical System

**Figure 1:**
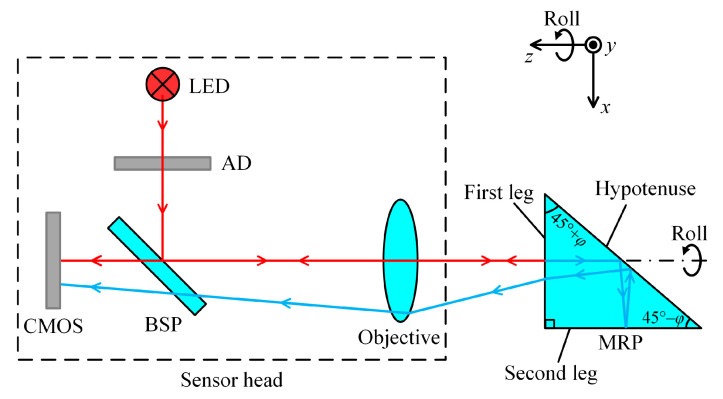
Optical system of roll angle sensor.

Figure 1 shows an optical system of a roll angle sensor. A light-emitting diode (LED) radiates a light beam toward an aperture diaphragm (AD). The beam through the central hole is reflected by a beam splitting plate (BSP) and collimated by an objective. When the collimated beam proceeds to the first leg (deposited with a beam splitting coating) of the MRP, it is separated into two beams. One beam is reflected towards the sensor head and converges into a light spot. Another beam transmits into the MRP and is sequentially reflected three times by the hypotenuse, second leg, and hypotenuse again (both the hypotenuse and second leg are deposited with a high reflection coating), and then refracted once more by the first leg. Similarly, this beam returns to the sensor head and converges into another light spot. A photodetector (CMOS) is fixed on the focal plane of the objective to collect the two light spots and convey them to a computer using a USB cable. The centroid coordinates of both light spots can be obtained using an image processing program.

### 2.2. Measuring Roll Angle

**Figure 2:**
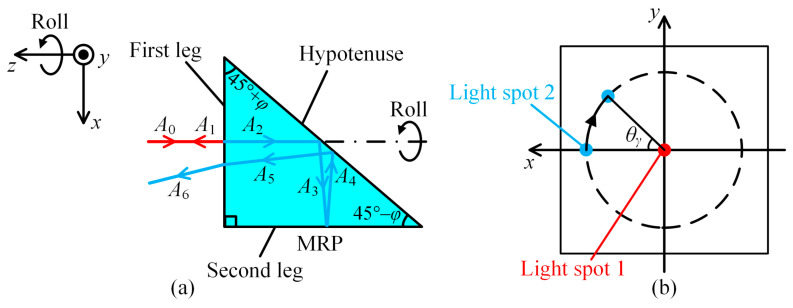
Measuring roll angle: (**a**) schematic diagram; (**b**) moving tracks of two light spots collected by CMOS.

Figure 2a illustrates the method for measuring the roll angle. In the space coordinate system, the roll is considered to be the rotation around the *z*-axis. The MRP is designed with one right angle and two acute angles (45° + *φ* and 45° − *φ*). In the initial state, no roll angle occurs. A collimated beam with orientation vector *A*_0_ = [0 0 −1]^T^ hits the first leg of the MRP and is split into a reflected beam and a transmitted beam. Complying with the law of reflection, the orientation vector of the reflected beam is calculated as follows:

(1) $$A_{1}=A_{0}-2\left(A_{0}\cdot I_{1}\right)I_{1}={\left[\begin{array}{ccc} 0 & 0 & 1 \end{array}\right]}^{T},$$

where *I*_1_ = [0 0 −1] represents the normal vector of the first leg. This beam then converges into light spot 1 in the middle of the CMOS, as indicated by the red dot in Figure 2b. Complying with the law of refraction, the orientation vector of the transmitted beam is calculated as follows:

(2) $$A_{2}=A_{0}+\left[\sqrt{n^{2}-1+{\left(A_{0}\cdot I_{1}\right)}^{2}}-A_{0}\cdot I_{1}\right]I_{1}={\left[\begin{array}{ccc} 0 & 0 & -n \end{array}\right]}^{T},$$

where *n* represents the refractive index of the MRP. This beam is then sequentially reflected by the hypotenuse, second leg, and hypotenuse again. The orientation vectors of the beams reflected from each surface are calculated as follows:

(3) $$\left\{\begin{array}{l} A_{3}=A_{2}-2\left(A_{2}\cdot I_{2}\right)I_{2}={\left[\begin{array}{ccc} n & 0 & -2n\varphi \end{array}\right]}^{T},\\ A_{4}=A_{3}-2\left(A_{3}\cdot I_{3}\right)I_{3}={\left[\begin{array}{ccc} -n & 0 & -2n\varphi \end{array}\right]}^{T},\\ A_{5}=A_{4}-2\left(A_{4}\cdot I_{2}\right)I_{2}={\left[\begin{array}{ccc} 4n\varphi & 0 & n \end{array}\right]}^{T}, \end{array}\right.$$

where *I*_2_ = [–sin(45° + *φ*) 0 –cos(45° + *φ*)]^T^ and *I*_3_ = [1 0 0]^T^ represent the normal vectors of the hypotenuse and second leg, respectively. Subsequently, the beam with orientation vector *A*_5_ is refracted by the first leg, and the orientation vector of the transmitted beam is calculated as follows:

(4) $$A_{6}=A_{5}+\left\{\sqrt{1-n^{2}+{\left[A_{5}\cdot \left(-I_{1}\right)\right]}^{2}}-A_{5}\cdot \left(-I_{1}\right)\right\}\left(-I_{1}\right)={\left[\begin{array}{ccc} 4n\varphi & 0 & 1 \end{array}\right]}^{T}.$$

Eventually, this beam converges into light spot 2 on the CMOS, as indicated by the blue dot in Figure 2b. If the MRP generates a roll angle *θ_γ_* with a measured target, the rotation matrix can be written as follows:

(5) $$M=\left[\begin{array}{ccc} \cos\theta_{\gamma } & -\sin\theta_{\gamma } & 0\\ \sin\theta_{\gamma } & \cos\theta_{\gamma } & 0\\ 0 & 0 & 1 \end{array}\right].$$

Accordingly, the normal vectors of each surface of the MRP change to the following:

(6) $$\left\{\begin{array}{l} {I_{1}}^{\prime }=MI_{1}={\left[\begin{array}{ccc} 0 & 0 & -1 \end{array}\right]}^{T},\\ {I_{2}}^{\prime }=MI_{2}=-\frac{\sqrt{2}}{2}{\left[\begin{array}{ccc} \left(1+\varphi \right)\cos\theta_{\gamma } & \left(1+\varphi \right)\sin\theta_{\gamma } & \left(1-\varphi \right) \end{array}\right]}^{T},\\ {I_{3}}^{\prime }=MI_{3}={\left[\begin{array}{ccc} \cos\theta_{\gamma } & \sin\theta_{\gamma } & 0 \end{array}\right]}^{T}. \end{array}\right.$$

Following the above calculation process, the orientation vector of the beam reflected by the first leg of the MRP is calculated as follows:

(7) $${A_{1}}^{\prime }={\left[\begin{array}{ccc} 0 & 0 & 1 \end{array}\right]}^{T}.$$

The orientation vector of the beam that withstands multiple refractions and reflections by the MRP is calculated as follows:

(8) $${A_{6}}^{\prime }={\left[\begin{array}{ccc} 4n\varphi \cos\theta_{\gamma } & 4n\varphi \sin\theta_{\gamma } & 1 \end{array}\right]}^{T}.$$

Through a comparison between Equations (1), (4), (7) and (8), the coordinates of light spots 1 and 2 can be written as follows:

(9) $$\left\{\begin{array}{c} x_{1}=0,\\ y_{1}=0, \end{array}\right.\mathrm{and}\;\left\{\begin{array}{c} x_{2}=4fn\varphi \cos\theta_{\gamma },\\ y_{2}=4fn\varphi \sin\theta_{\gamma }. \end{array}\right.$$

Figure 2b shows the moving tracks of the two light spots collected by the CMOS before and after the MRP rolls. Light spot 1 remains stationary in the middle of the CMOS, while light spot 2 revolves around it by a central angle of *θ_γ_* and a track radius of 4*fnφ*. Therefore, it can be summarized that the roll angle of the MRP equals the rotation angle of the line segment defined by the two light spots, which can be expressed as in the following equation:

(10) $$\theta_{\gamma }=\arctan\left(y_{2}/x_{2}\right).$$

### 2.3. Crosstalk Effect

The roll of a measured target may be accompanied by its pitch and yaw, that is, crosstalk occurs. The crosstalk of the pitch and yaw to the roll is discussed in this subsection. If the MRP rotates around the three coordinate axes with the measured target, the rotation matrix can be recast as follows:

(11) $$M^{\prime }=\left[\begin{array}{ccc} 1 & 0 & 0\\ 0 & \cos\theta_{\alpha } & -\sin\theta_{\alpha }\\ 0 & \sin\theta_{\alpha } & \cos\theta_{\alpha } \end{array}\right]\left[\begin{array}{ccc} \cos\theta_{\beta } & 0 & \sin\theta_{\beta }\\ 0 & 1 & 0\\ -\sin\theta_{\beta } & 0 & \cos\theta_{\beta } \end{array}\right]\left[\begin{array}{ccc} \cos\theta_{\gamma } & -\sin\theta_{\gamma } & 0\\ \sin\theta_{\gamma } & \cos\theta_{\gamma } & 0\\ 0 & 0 & 1 \end{array}\right],$$

where *θ_α_* and *θ_β_* represent the pitch angle and yaw angle, respectively, with their values being small. Accordingly, the normal vectors of each surface of the MRP change to the following:

(12) $$\left\{\begin{array}{l} {I_{1}}^{\prime \prime }=M^{\prime }I_{1}={\left[\begin{array}{ccc} -\theta_{\beta } & \theta_{\alpha } & -1 \end{array}\right]}^{T},\\ {I_{2}}^{\prime \prime }=M^{\prime }I_{2}=-\frac{\sqrt{2}}{2}{\left[\begin{array}{ccc} \left(1+\varphi \right)\cos\theta_{\gamma }+\theta_{\beta } & \left(1+\varphi \right)\sin\theta_{\gamma }-\theta_{\alpha } & \theta_{\alpha }\sin\theta_{\gamma }-\theta_{\beta }\cos\theta_{\gamma }+\left(1-\varphi \right) \end{array}\right]}^{T},\\ {I_{3}}^{\prime \prime }=M^{\prime }I_{3}={\left[\begin{array}{ccc} \cos\theta_{\gamma } & \sin\theta_{\gamma } & \theta_{\alpha }\sin\theta_{\gamma }-\theta_{\beta }\cos\theta_{\gamma } \end{array}\right]}^{T}. \end{array}\right.$$

Still following the above calculation process, the orientation vector of the beam reflected by the first leg of the MRP is calculated as follows:

(13) $${A_{1}}^{\prime \prime }={\left[\begin{array}{ccc} 2\theta_{\beta } & -2\theta_{\alpha } & 1 \end{array}\right]}^{T}.$$

The orientation vector of the beam subjected to multiple refractions and reflections by the MRP is calculated as follows:

(14) $${A_{6}}^{\prime \prime }={\left[\begin{array}{ccc} 4n\varphi \cos\theta_{\gamma }+2\theta_{\beta } & 4n\varphi \sin\theta_{\gamma }-2\theta_{\alpha } & 1 \end{array}\right]}^{T}.$$

According to Equations (13) and (14), the coordinates of light spots 1 and 2 can be recast as follows:

(15) $$\left\{\begin{array}{l} x_{1}=2f\theta_{\beta },\\ y_{1}=-2f\theta_{\alpha }, \end{array}\right.\mathrm{and}\;\left\{\begin{array}{l} x_{2}=4fn\varphi \cos\theta_{\gamma }+2f\theta_{\beta },\\ y_{2}=4fn\varphi \sin\theta_{\gamma }-2f\theta_{\alpha }. \end{array}\right.$$

**Figure 3:**
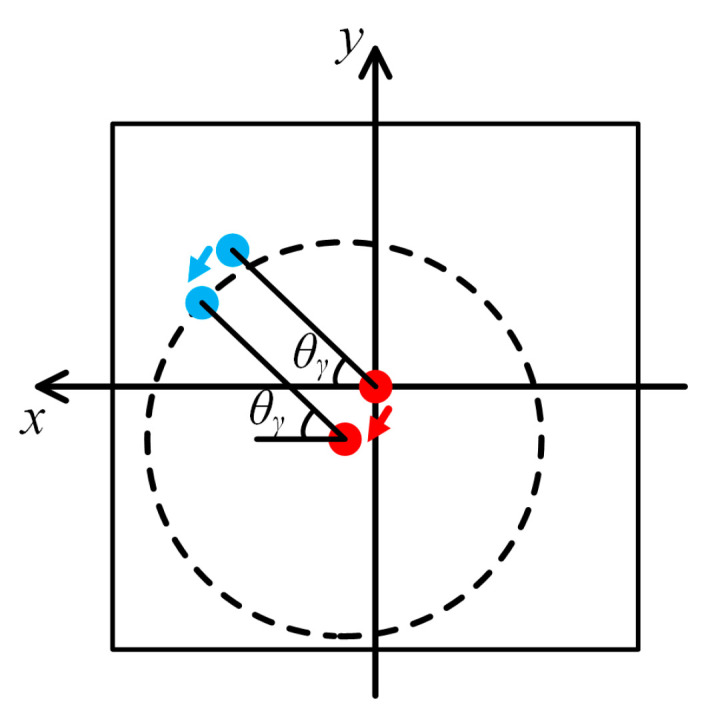
Moving tracks of two light spots collected by CMOS under crosstalk.

A comparison between Equations (9) and (15) indicates that the pitch and yaw translate the two light spots along the same direction by the same amount, as shown in Figure 3. Nevertheless, light spot 2 still rotates around light spot 1 by a central angle of *θ_γ_*. Thus, the roll angle of the MRP can be expressed as in the following:

(16) $$\theta_{\gamma }=\arctan\left[\left(y_{2}-y_{1}\right)/\left(x_{2}-x_{1}\right)\right].$$

When Equation (16) is used to calculate the roll angle, the translations of the two light spots are completely counteracted. As a result, the pitch and yaw do not interfere with the roll angle measurement.

## 3. Experimental Results

### 3.1. Experimental Apparatus

**Figure 4:**
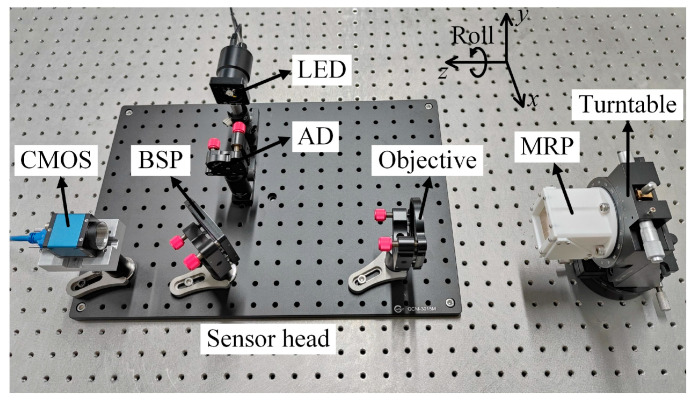
Photograph showing experimental apparatus to test the proposed roll angle sensor.

Figure 4 shows the experimental apparatus used to evaluate the validity of the proposed sensor for measuring the roll angle. The LED (Daheng Optics GCI-060401, Beijing, China) had a 620 nm central wavelength and 3 W output power. The CMOS (Daheng Imaging ME2P-2621-15U3M, Beijing, China) had a 2.5 μm pixel size and contained 5120 × 5120 pixels. The AD had a circular hole with a 2 mm diameter. The BSP had a 50% splitting ratio. The objective had 300 mm focal distance and 50.8 mm diameter. The dimensions of the MRP were 40 × 40 × 40.2334 mm^3^ (product of side lengths of two legs), forming two acute angles of 45°10′ and 44°50′. Note that the tolerance of the angle *φ* of the MRP was –20 to –10 arcsec. The substrate material of the MRP was N-BK7 glass with a 1.517 refractive index. Consequently, the angles of the two outgoing beams returned by the MRP did not exceed 0.9945° relative to the *z*-axis. In addition, the MRP had a beam splitting coating (62.3% transmissivity) on the first leg and a high reflection coating (97.5% reflectivity) on the second leg and hypotenuse, respectively. Thus, the grayscale distributions of the two light spots on the CMOS were approximately the same, which suppressed the positioning error caused by the grayscale discrepancy. Both light spots collected by the CMOS were sent to a computer for preprocessing. Subsequently, a gray centroid algorithm with a power of two was used to extract the centroid coordinates of each light spot, which can be expressed as follows:

(17) $$x_{c}=\frac{\sum_{i=1}^{u}\sum_{j=1}^{v}i\times g^{2}\left(i,j\right)}{\sum_{i=1}^{u}\sum_{j=1}^{v}g^{2}\left(i,j\right)},\;\;y_{c}=\frac{\sum_{i=1}^{u}\sum_{j=1}^{v}j\times g^{2}\left(i,j\right)}{\sum_{i=1}^{u}\sum_{j=1}^{v}g^{2}\left(i,j\right)},$$

where *i* and *j* represent the abscissa and ordinate of a pixel in an image of size *u* × *v*, respectively, and *g*(*i*, *j*) represents the gray value of the pixel. Ultimately, the roll angle of the MRP can be calculated by substituting the centroid coordinates of both light spots into Equation (16).

### 3.2. Stability and Repeatability Tests

**Figure 5:**
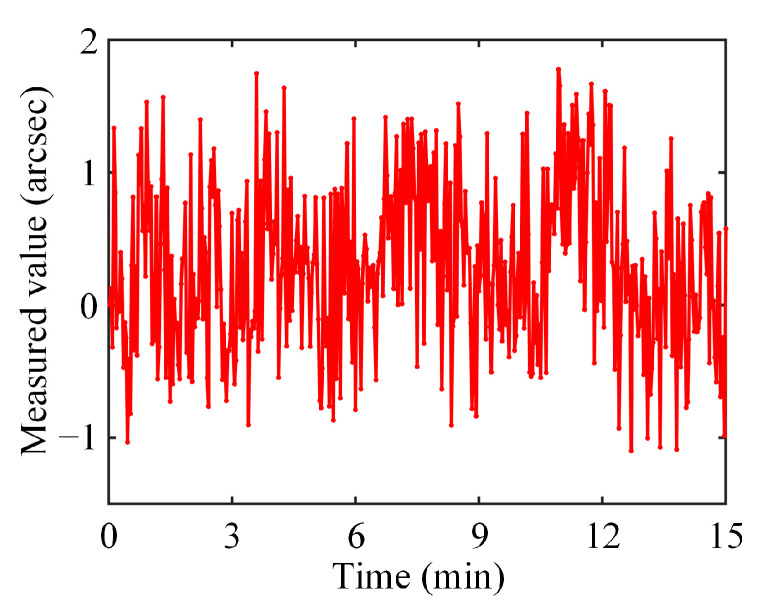
Measured curve of roll angle of MRP.

The reliability of the measurement depends on the stability of the sensor. Therefore, the first task after building the roll angle sensor was to test its stability. The test was operated on an optical stage in a laboratory to isolate mechanical vibrations. The temperature and humidity of the laboratory were controlled at 25 °C and 35%, respectively, and there was no illumination. The MRP was screwed onto a three-axis turntable in front of the sensor head. The turntable was rotated such that the angle between the line segment defined by the two light spots and the *x*-axis was approximately 87°, and light spot 1 was situated in the middle of the CMOS. Half an hour after the roll angle sensor was switched on, the CMOS captured one picture every second for a total duration of 15 min. Figure 5 shows the measured curve of MRP’s roll angle. The peak-to-valley value of the curve was 2.88 arcsec, and the standard deviation was 0.62 arcsec. The primary cause of the curve fluctuation was random errors, including electronic noise inside the CMOS, the jitter of the LED output power, and interference from the environment. Nevertheless, the overall trend of the measured curve indicated that the proposed sensor was stable.

Additionally, a repeatability test was performed. During the stable measurement, a measured value of the roll angle was recorded as the initial value. Then, a light barrier was placed between the sensor head and the MRP and removed after a few seconds. At this time, another measured value (deviation from the initial value) was recorded. The above operation was repeated nine times; the measured values were −0.27, 0.03, 0.59, 0.16, −0.68, 0.22, −0.08, 0.28, and −0.4 arcsec, respectively, and the standard deviation was calculated to be 0.39 arcsec according to the Bessel formula, indicating fine repeatability.

### 3.3. Roll Angle Calibration

**Figure 6:**
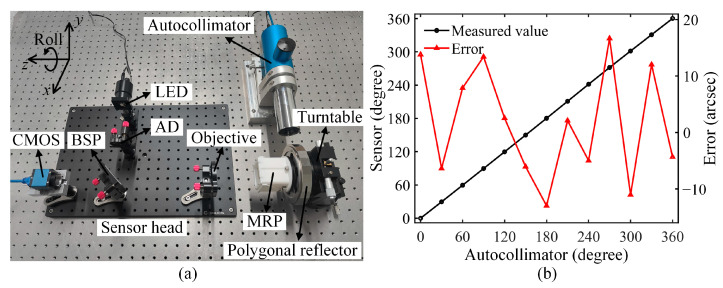
Calibration of roll angle: (**a**) experimental setup and (**b**) results.

To calibrate the proposed roll angle sensor, a two-axis photoelectric autocollimator and a polygonal reflector were used as references. The polygonal reflector had 24 reflective surfaces, and the included angle between the normals of any two contiguous surfaces was 15°. The autocollimator (Shanghai MicroCre Light-Machine YRMAT-2010B, Shanghai, China) had a 0.1 arcsec resolution and a ±0.5 arcsec accuracy. Figure 6a shows the calibration setup. The MRP and polygonal reflector were fastened to a turntable and could roll synchronously. Firstly, the pitch and yaw angles of the turntable was fine-tuned such that light spot 1 was located in the central zone of the CMOS. Secondly, the turntable was rolled by a full circle (360°) while light spot 2 was observed to determine whether it would exceed the CMOS photosensitive area. If it did, the two above-mentioned steps were repeated to make sure that light spot 2 was always within the photosensitive area and that the distances between the centroid of light spot 2 and the four sides of the photosensitive area were approximately equal when light spot 2 was closest to the four sides. In this way, the sensor head was aligned perpendicularly to the first leg of the MRP. The autocollimator on the side was aligned vertically to one reflective surface of the polygonal reflector. Then, the turntable was rolled in 30° increments, and its roll angle was detected using the proposed sensor and autocollimator. Figure 6b shows the calibration results. Comparing the measured values given by the proposed sensor with the reference values offered by the autocollimator resulted in a series of measurement errors, which varied within ±13.85 arcsec with a standard deviation of 9.75 arcsec over a range of 360°. Contributors to the measurement errors included random errors, as discussed in the previous subsection, and systematic errors such as the flatness errors on the two legs and one hypotenuse of the MRP, non-parallelism of its crest lines owing to manufacturing defects, and mechanical deformation owing to the residual stress during assembly. A linear fit was also performed on the measured values of the proposed sensor and a linearity of 0.9969 was obtained.

### 3.4. Comparison and Resolution Tests

**Figure 7:**
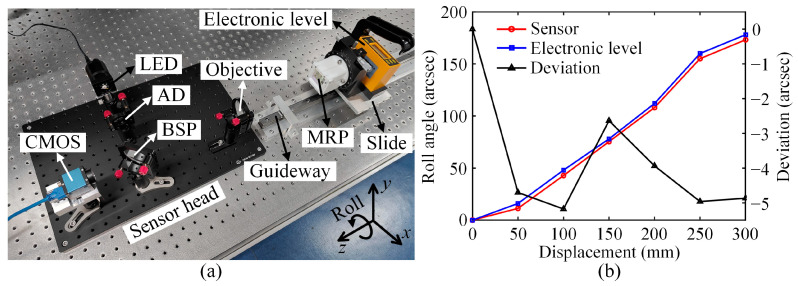
Comparison test: (**a**) experimental setup and (**b**) results.

In mechanical engineering, obtaining the roll angle of a guideway is often required to correct its profile. Therefore, a comparison test was conducted to appraise the performance of the proposed roll angle sensor in this practical measurement. Figure 7a shows the experimental setup. The sensor head and a linear guideway were screwed onto an optical stage. The MRP and an electronic level (Qingdao Qianshao WL9, Qingdao, China) with an accuracy of ±(1 + 0.02 × reading) were fixed to a slide which was manually slid along the guideway in the direction away from the sensor head, with intervals of 50 mm and a stroke of 300 mm. Then, the roll angle of the slide at different points of the guideway could be measured synchronously by the proposed sensor and electronic level. Figure 7b plots the measurement results. The measured curves of both devices exhibited good agreement, which verified that the proposed sensor could be used for practical measurements. The deviations acquired by deducting the reference values given by the electronic level from the measured values offered by the proposed sensor were better than −5.18 arcsec. The main causes of these deviations might be the spherical aberration of the objective, as well as the misorientation of the electronic level and the different assembly stresses released by it and the MRP. Incidentally, the pitch and yaw angles of the MRP can be measured simply through the light spot displacement of the beam reflected by the first leg of the MRP according to the traditional autocollimation principle. Therefore, the proposed sensor can also be used for the simultaneous measurement of three-dimensional angles.

**Figure 8:**
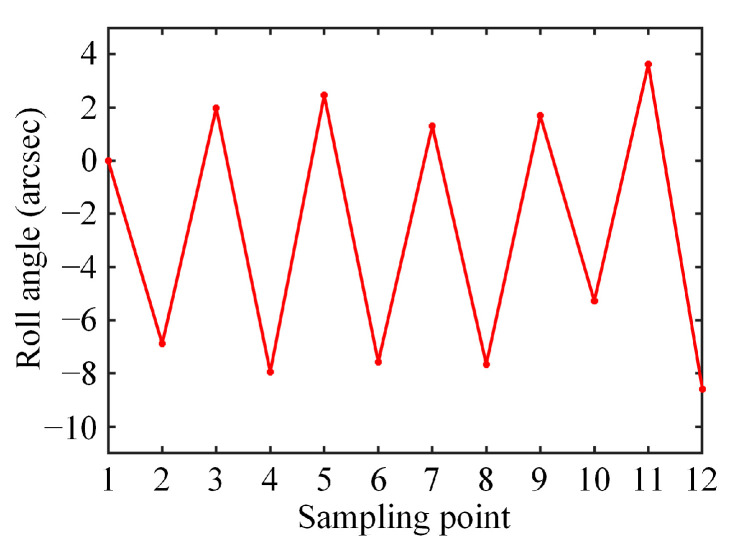
Resolution test results.

Furthermore, a resolution test was performed based on this experimental setup. The slide carrying the MRP and the electronic level was located approximately 50 mm from the sensor head. Several small steel plates were stacked on one side of the slide to force it to roll slightly (overseen by the electronic level), and then these plates were taken away. This operation was repeated multiple times, and the proposed sensor was used to measure the roll angle of the slide during this period. Figure 8 shows the measurement results. Each data point was the arithmetic mean of ten measurements. As can be seen, the proposed sensor could recognize reciprocating changes of 10 arcsec, thus the roll angle resolution reached at least 10 arcsec.

## 4. Conclusions and Discussion

This paper presents a novel roll angle sensor based on the autocollimation principle, which can measure the roll angle within 360°. The MRP is exquisitely designed to function as a reflector to admit the collimated beam and return two outgoing beams to the sensor head, and then both beams would converge into two light spots on the CMOS. The theoretical derivation demonstrates that the roll angle of the MRP is equivalent to the rotation angle of the line segment defined by the two light spots on the CMOS, which can be determined by substituting the centroid coordinates of both light spots into the established mathematical model. The calibration results show that the measurement accuracy of the proposed sensor achieved ±13.85 arcsec over a range of 360°, thus it can be used for roll angle measurements that require a large measuring range.

Despite the significant results, further studies are necessary. First, the measurement error should be reduced. The random error, as one of the contributors, can be decreased by introducing a filtering algorithm or correction circuit and by improving the working temperature and sampling parameters of the CMOS. The systematic error, as another one of the contributors, can be diminished through intensive calibration, error curve fitting, and compensation. Second, the resolution of the roll angle needs to be enhanced, which can be materialized by increasing the focal distance of the objective, the refractive index and angle *φ* of the MRP, and by reforming the centroid extraction algorithm of the light spot. Third, the proposed sensor should be miniaturized to accommodate more application scenarios and increase its portability. The main factors hindering miniaturization include the size of the components used and the focal distance of the objective. For the former, the components used can be replaced with smaller sized ones as long as they do not block the light beam. For the latter, multiple mirrors can be introduced to fold the optical path to reduce the overall size. Reducing the measurement error, improving the roll angle resolution, and miniaturizing the proposed sensor will be the focus of future studies.

## Data Availability

Data are contained within the article.
